# Psychological Reactions of Hospital Workers to a Pandemic: A Comparison of SARS-CoV-2 in 2020 and SARS in 2003

**DOI:** 10.3390/ijerph19020833

**Published:** 2022-01-12

**Authors:** Yu Lee, Liang-Jen Wang, Wen-Jiun Chou, Ming-Chu Chiang, Shan Huang, Yi-Chun Lin, Jie-Yi Lin, Nien-Mu Chiu, Chih-Hung Chen, Ing-Kit Lee, Chia-Te Kung, Chih-Chi Wang, Mian-Yoon Chong

**Affiliations:** 1 Department of Psychiatry, Kaohsiung Chang Gung Memorial Hospital and Chang Gung University College of Medicine, Kaohsiung 83301, Taiwan; lyu722@cgmh.org.tw (Y.L.); neinmuch@cgmh.org.tw (N.-M.C.); 2 Department of Child and Adolescent Psychiatry, Kaohsiung Chang Gung Memorial Hospital and Chang Gung University College of Medicine, Kaohsiung 83301, Taiwan; wjchou@adm.cgmh.org.tw; 3 Department of Nursing, Kaohsiung Chang Gung Memorial Hospital, Kaohsiung 83301, Taiwan; e2988386@cgmh.org.tw; 4 Administrative Offices, Kaohsiung Chang Gung Memorial Hospital, Kaohsiung 83301, Taiwan; t70726@adm.cgmh.org.tw (S.H.); ddt766@cgmh.org.tw (Y.-C.L.); jy866@cgmh.org.tw (J.-Y.L.); 5 Division of General Medicine, Department of Internal Medicine, Kaohsiung Chang Gung Memorial Hospital and Chang Gung University College of Medicine, Kaohsiung 83301, Taiwan; totoro@cgmh.org.tw; 6 Division of Infectious Disease, Department of Internal Medicine, Kaohsiung Chang Gung Memorial Hospital and Chang Gung University College of Medicine, Kaohsiung 83301, Taiwan; leee@cgmh.org.tw; 7 Department of Emergency, Kaohsiung Chang Gung Memorial Hospital and Chang Gung University College of Medicine, Kaohsiung 83301, Taiwan; g00308@cgmh.org.tw; 8 Division of General Surgery, Department of Surgery, Kaohsiung Chang Gung Memorial Hospital and Chang Gung University College of Medicine, Kaohsiung 83301, Taiwan; ufel4996@ms26.hinet.net

**Keywords:** psychological reactions, SARS-CoV-2, SARS, hospital workers

## Abstract

Epidemic viral infections, including the outbreak of severe acute respiratory syndrome (SARS) in 2003 and SARS-CoV-2 in 2019, have brought tremendous loss to people across the nations. The aim of this study was to compare the psychological impact of the SARS-CoV-2 pandemic in 2020 and the SARS pandemic in 2003 on hospital workers. Hospital workers at a medical center in Southern Taiwan (*n* = 1816) were invited to complete questionnaires (SARS-CoV-2 Exposure Experience, the Impact of Event Scale, the Chinese Health Questionnaire, and the Distress Thermometer). The current data were compared to the data collected from hospital workers (*n* = 1257) at the same medical center during the SARS pandemic in 2003. We found the psychological impact on hospital workers during the SARS-CoV-2 pandemic was significantly lower than that during the previous SARS period. During the SARS-CoV-2 pandemic period, hospital workers with SARS experience were more accepting of the risk, felt a greater responsibility to take care of the SARS-CoV-2 patients, and were more likely to perceive the danger of becoming infected. The associated factors of psychiatric morbidity in hospital workers with SARS experience were being female, the degree of intrusion severity, and severity of psychological distress. Proper management strategies and lessons learned from the SARS experience might have led to low psychiatric morbidity during the SARS-CoV-2 pandemic.

## 1. Introduction

Viruses have no national boundary and since ancient times, if they have spiraled out of control, they have become epidemic and even pandemic. Sometimes, pandemics occur when new diseases, such as the Black Death epidemic of bubonic plague, develop the ability to spread rapidly [1]. In the last decades, epidemic viral infections, including severe acute respiratory syndrome (SARS) in 2003, influenza caused by the virus subtype H1N1 in 2009, Middle East Respiratory Syndrome (MERS) in 2012, Ebola virus disease in 2014, and SARS-CoV-2 in 2019, have brought tremendous loss to humans across the world [2,3,4].

A pandemic can lead to more social disruption, economic loss, and general hardship than an epidemic. Moreover, the psychological impact of a pandemic virus infection may spread to the community and even to hospital workers due to the infection’s high morbidity and mortality [5]. Hospital workers have to deal with an invisible enemy that might induce sleep problems, a psychosomatic reaction, anxiety, or depression [6,7]. A study revealed that dealing with SARS-CoV-2 led to high levels of distress in approximately 30% of primary care physicians in China [8]. Of all the viral infectious diseases that occurred in the last decade, SARS-CoV-2 is the most infectious and the longest lasting and has caused huge losses, including to the economy, social relationships, and life itself [9,10]. In dealing with the SARS-CoV-2 pandemic outbreak, challenges for the staff at hospitals include not only the increased workload created by such outbreaks, but also fears of contagion to themselves and the burden of caring for patients who are very sick [11,12]. Hospital workers are at risk of having psychological reactions and developing other mental health symptoms [12].

Prior to the SARS-CoV-2 pandemic in 2019, the severe acute respiratory syndrome (SARS) epidemic in 2003 had a huge negative impact on people’s health across the world. At that time, SARS was a major international public health problem, with 8096 probable cases and 774 deaths worldwide from 1 November 2002 to 31 July 2003 [13]. The inherent scientific uncertainties and evolving nature of the SARS outbreaks caused considerable panic and fear, mainly in East Asia, because of the rapid transmission and high mortality rate of the disease [14]. The life-threatening potential of occupational exposure to SARS was a very important concern faced by healthcare workers [15].

In late April 2003, the first case of SARS was admitted to the Kaohsiung Chang Gung Memorial Hospital, a tertiary hospital in southern Taiwan. A few days later, nosocomial infection occurred, resulting in 79 infections and 19 deaths. Among the infected were 16 hospital staff members (5 doctors, 9 nurses, and 2 respiratory therapists) who were also kept in the intensive care unit; unfortunately, one of them (a doctor) died on 16 May 2003. Moreover, a total of 237 hospital staff members were mandatorily quarantined for 14 days in the new isolation dormitory because of close contact with an infected person. Starting from mid-May, measures to control nosocomial infection were strictly implemented: all outpatient and emergency services were closed and new admissions were not accepted; the number of staff on duty was consequently reduced [16]. At that time, senior psychiatrists, along with their teams of residents, psychologists, and social workers, provided hospital workers at different nursing stations with a total of 14 single debriefing sessions designed for stress management. The psychiatric department also conducted a questionnaire survey assessing the psychological impact of the SARS pandemic on hospital workers and found that most of the staff experienced somatic symptoms and anxiety during the initial phase of SARS and felt depressed during the repair phase [16].To date, very few studies have focused on the psychological reactions, including psychological impact, distress, and psychiatric morbidity, of hospital workers who had or had no exposure to SARS prior to the outbreak of SARS-CoV-2. Although SARS-CoV-2 and SARS both caused global pandemics, the two pandemic episodes have two different contexts. SARS was a highly fatal (mortality rate of around 20%) and fast-fading disease; SARS-CoV-2 is a less fatal (mortality of around 2%) [17], but more persistent pandemic disease. Therefore, the main psychological repercussion (impact) of SARS is post-traumatic stress symptoms [14,15,16], whereas the psychological impacts of SARS-CoV-2 are post-traumatic stress symptoms, insomnia, anxiety, and depression [18,19]. Considering the fact that both of these pandemics followed a different course and based on the Kübler-Ross model, the psychological mechanisms of SARS are exhibited in the denial and anger stages, with the symptoms of shock, apprehension, and anger; the psychological mechanisms of SARS-CoV-2 are seen in the bargaining and depression stage, with the symptoms of anxiety, insomnia, and dysphoria [20].

We hypothesized that the psychological impact on hospital workers during the SARS-CoV-2 pandemic might be different from that observed during the SARS outbreak. In addition, based on the “psychological trauma and risk awareness” hypothesis (i.e., psychological trauma due to natural disasters is related to future preparedness and risk awareness), we hypothesized that the psychological impact on hospital workers who had exposure to SARS would be lower than on those without exposure to SARS. The study aimed to compare the psychological impact of SARS-CoV-2 and SARS on hospital workers at the same general hospital. In addition, this study proposed to compare the psychological reactions of hospital workers during the SARS-CoV-2 period to those of hospital workers at the same hospital who had or had no exposure to SARS in 2003.

## 2. Materials and Methods

### 2.1. Background of the Study

In Taiwan, the first identified case of SARS-CoV-2 was reported on 21 January 2020, but an outbreak did not really ensue. By 15 May 2021, there were 1475 cases, and 12 deaths had occurred. Among the 1475 cases, 1078 were imported, 344 were local, 36 were from a Fleet of Friendship, 2 were from aircrafts, 14 were under investigation, and the origin of one case has yet to be determined.

Kaohsiung Chang Gung Memorial Hospital (KCGMH), a tertiary care hospital in southern Taiwan with 2754 beds and 6204 workers (including ancillary staff), admitted its first patient with SARS-CoV-2 on 15 March 2020. In all, 147 suspected cases due to SARS-CoV-2 have been isolated at KGCMH; of these, only 9 individuals (5 males and 4 females), with an average age of 35.2 (19–62) years, were confirmed to have SARS-CoV-2 and were admitted to KCGMH. The average admission duration was 39.7 (24–76) days. No health worker has been infected.

### 2.2. Study Design and Measures

This study is a cross-sectional survey. All staff on service during the outbreak were invited to participate. The study covered one month (from 30 June 2020 to 31 July 2020). Data were collected through an anonymous, self-assessed questionnaire distributed to all workstations via the Internet (all hospital staff can access it for free). The study project was approved by the human research ethics committee of Chang Gung Memorial Hospital (CGMH) (202000805B0C601). Since participation was voluntary and survey responses were anonymous, the Institutional Review Board (IRB) of CGMH ruled that this study did not require informed consent. The questionnaire was composed of four parts: basic demographic data and SARS-CoV-2 exposure experience, the Impact of Event Scale (IES), the Chinese-version Health Questionnaire (CHQ), and the Distress Thermometer (DT).

In order to compare psychological reactions during both pandemics (SARS and SARS-CoV-2) among the same hospital workers, we used the same methods, including anonymous self-rated questionnaires, and recruited all hospital workers in both studies. All of the questionnaires, with the exception of the DT, were the same as those used in prior studies during SARS in order to compare the exposure experience, psychological impact, and psychiatric morbidity of hospital workers during two different periods at the same hospital [16]. Another difference between the two studies is that all participants in the SARS-CoV-2 study completed the questionnaires using free-access Internet, whereas nearly 60% of the participants during the SARS study responded using the pencil-and-paper method and 40% participated using the Internet. About half of participants in the SARS study responded, while only one third of participants in the SARS-CoV-2 study responded.

#### 2.2.1. Exposure to SARS-CoV-2

Information about exposure to SARS-CoV-2 and work experience was collected, including an assessment of the nature and location of the work as well as the contact with and care of SARS-CoV-2 patients. In addition, items related to the perception of risk, unfavorable experiences, and individual responses were evaluated using a five-point Likert scale (1, strongly disagree; 2, disagree; 3, uncertain; 4, agree; 5, strongly agree). This SARS-CoV-2 Exposure Experience Scale was based on a previous SARS Exposure Experience Scale, with the author’s approval [16].

#### 2.2.2. Impact of Event Scale (IES)

The Impact of Event Scale, a self-reporting measure, has been widely used in different populations and with a variety of traumas in order to assess the impact of specific stressful life events [21]. Each of the 15 items on the list was scored on a four-point frequency scale related to the past week (0, not at all; 1, rarely; 3, sometimes; 5, often). The higher the score, the higher the frequency of intrusive thoughts and avoidance attempts. The IES has been translated into Chinese and has shown satisfactory effectiveness in a study of oral cancer patients [22]. The IES has also been used to assess adolescent victims of an earthquake in Taiwan [23]. Chen (2005) used the IES to assess the psychological distress of nurses who worked during the outbreak of SARS in Taiwan [15]. As for SARS-CoV-2, IES was utilized to assess the possible psychological trauma of participants in the study [24].

#### 2.2.3. Chinese-Version Health Questionnaire (CHQ)

The culturally sensitive Chinese-version Health Questionnaire is a self-managed screening tool used to assess mental illness in the Chinese ethnic community [25]. The CHQ is derived from the general health questionnaire (GHQ) [26] and has been verified to have satisfactory structural validity, and it has been applied to the investigation of the incidence of mental illness in the community [25] and the hospital environment [27]. The CHQ includes four factors: physical symptoms, anxiety and worry, sleep problems, and depression and poor family relationships [28]. In this study, the 12-item version of CHQ-12 was used. The cut-off point of 2/3 was adopted for case/non-case, as used in the community study.

#### 2.2.4. Distress Thermometer (DT)

The DT is a single-item self-reporting screening tool used to assess the psychological distress of cancer patients [29,30]. The DT grades the degree of distress during the previous week based on a visual analog scale, with a score ranging from 0 (indicating no distress) to 10 (indicating extreme distress). The DT has been used in different populations and its performance has been compared with different self-reported symptom scales [31].

### 2.3. Statistical Analysis

Comparison of the psychiatric morbidity and psychological impact of two different pandemics (SARS-CoV-2 and SARS) on hospital workers was first originated by means of the chi-square and t tests. A one-way multivariate analysis of covariance (MANCOVA) was used to examine differences in psychological impact, psychological distress, psychiatric morbidity (dependent variables), and exposure experience to SARS-CoV-2 between hospital workers who had or had no exposure to SARS (independent variables), controlling for confounding factors (gender, age, work experience, living condition, job title, marital status, quarantine, and SARS-CoV-2 experience). Logistic regression was used to examine the perception of threat between hospital workers who had or had no exposure to SARS (independent variables), controlling for the aforementioned confounding factors. Finally, a stepwise forward model of logistic regression was used to test the risk factors associated with psychiatric morbidity (applying likelihood ratio estimation).

## 3. Results

### 3.1. Characteristics of Respondents

Of the 6204 workers eligible for the study, 2023 completed the questionnaire. Two hundred five respondents (10.1%) were excluded owing to their refusal to be analyzed. Of the 1816 successful respondents, 1544 (85.0%) were women and 272 (15.0%) were men, with a mean age of 38.7 (s.d. = 10.2) years (range 21–69 years). Most of the respondents were nurses (*n* = 1124; 61.9%), followed by health administrative workers/technicians (*n* = 560; 30.8%), and doctors (*n* = 132; 7.3%). Their length of work experience varied from less than 2 years to more than 44 years, with an average of 14.9 (s.d. = 10.2) years. Fifty-five percent of the respondents were married, and 76% were living with their families (Table 1).

### 3.2. Comparison of Clinical Characteristics of Hospital Workers during SARS-CoV-2 and SARS

Hospital workers during SARS-CoV-2 (*n* = 1816), compared to those during SARS (*n* = 1257), were more often female (85.0% vs. 81.1%), of older age (38.6 ± 10.3 years vs. 31.8 ± 6.43 years), part of the nursing staff (61.9% vs. 53.8%), married (55.2% vs. 49.4%), living with their families (76.3% vs. 65.6%), had longer work experience (14.8 ± 10.3 vs. 8.5 ± 5.7), spent less time taking care of patients (SARS-CoV-2/SARS) (4.3% vs. 9.9%), and spent less time in quarantine (4.1% vs. 6.3%) (Table 1). From 15 March to 29 May 2020, about one tenth (*n* = 185) of the respondents had been in contact with persons with the illness, while the rest were not sure (20.0%, *n* = 364) or had not been exposed (69.8%, *n* = 1267). Hospital workers during SARS-CoV-2 spent less time in quarantine and less time taking care of patients than hospital workers during SARS (Table 1).

### 3.3. Impact of Events and Psychiatric Morbidity during SARS-CoV-2 and SARS

**Figure 1:**
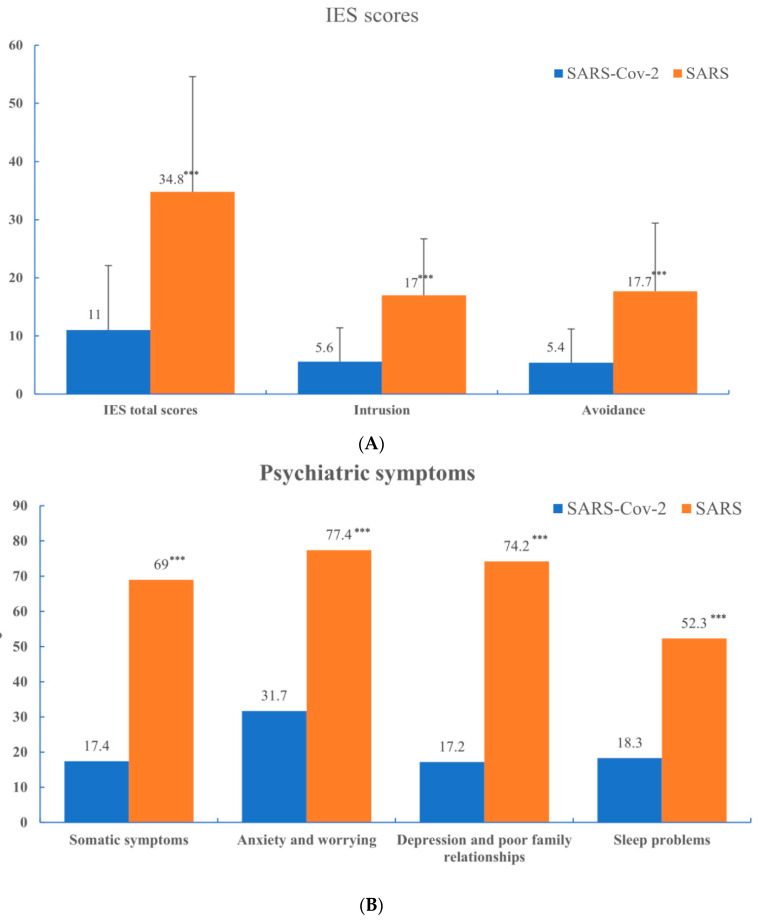
(**A**) Comparison of Impact of Event Scale scores in hospital workers during SARS-CoV-2 and SARS pandemics; (**B**) Comparison of psychiatric symptoms in hospital workers during SARS-CoV-2 and SARS pandemics *** *p* < 0.001.

The average IES score for our hospital workers during the SAS-Cov-2 pandemic was 11.0 (s.d. = 11.1). The IES scores (including total, intrusion, and avoidance) of our hospital workers during the SARS-CoV-2 pandemic were significantly lower than those of hospital workers during the SARS outbreak (Figure 1A). This result suggests that psychological impacts (include intrusive thoughts and avoidance attempts) were higher in hospital workers during the SARS outbreak than those of hospital workers during the SARS-CoV-2 pandemic.

Using the CHQ score as the case definition, the estimated prevalence of psychiatric morbidity in this sample (during the SARS-CoV-2 pandemic) was 21.1% (95% CI 19.3–23.0), which was lower than during the SARS outbreak (75.3%, 95% CI 72.9–77.7). The manifestations of psychiatric symptoms in this sample varied, with respondents reporting: 31.7%, anxiety and worrying; 18.3%, sleep problems; 17.4%, somatic symptoms; and 17.2%, depression and poor family relationships. The psychiatric symptoms (including anxiety and worrying, depression and poor family relationships, somatic symptoms, and sleep problems) of our hospital workers during the SARS-CoV-2 pandemic were significantly fewer than those of hospital workers during the SARS outbreak (Figure 1B).

### 3.4. Comparison of Characteristics and Psychological Impact of Hospital Workers Who Have Had or Not Had Exposure to SARS

Of the 1816 participants, all of whom had experience with SARS-CoV-2, 696 had experience with SARS and 1120 had no experience with SARS at our hospital. Hospital workers who had SARS experience were often male (18.0% vs. 13.1%), elderly (48.4 ± 6.2 vs. 32.6 ± 6.9), married (79.0% vs. 40.4%), living with their families (86.9% vs. 69.6%), more experienced workers (25.3 ± 6.10 vs. 8.3 ± 6.0), technicians/administrators (40.4% vs. 24.9%), and had undergone more quarantine (4.1% vs. 1.5%) (Table 2).

We compared hospital workers who had and had no exposure to SARS using MANCOVA. No differences in IES (total, intrusion, avoidance) scores, DT scores, and psychiatric morbidity were observed between hospital workers with or without exposure to SARS (Table 3).

### 3.5. Perception of Threat

There were some differences in reported perceptions and feelings between hospital workers who had or had no exposure to SARS. Hospital workers who had exposure to SARS were significantly more likely to perceive the danger of becoming infected, more accepting of the risk, and felt a responsibility to take care of the SARS-CoV-2 patients compared with hospital workers who had no exposure to SARS (Table 4).

### 3.6. Risk of Psychiatric Morbidity in Hospital Workers Who Had Exposure to SARS

In the univariate analyses of 696 hospital workers who had exposure to SARS, factors significantly associated with psychiatric morbidity (CHQ ≥ 3) included being female (88.6% vs. 80.1%, *p* = 0.01), having a younger age (32.6 ± 6.9 vs. 48.4 ± 6.2, *p* < 0.001), showing shorter work experience (8.3 ± 6.0 vs. 25.3 ± 6.1, *p* < 0.001), having a higher IES intrusion score (10.1 ± 6.4 vs. 4.8 ± 4.8, *p* < 0.001), expressing a higher IES avoidance score (8.7 ± 5.9 vs. 4.6 ± 4.9, *p* < 0.001), and reporting a higher DT score (4.1 ± 1.9 vs. 1.9 ± 1.6, *p* < 0.001) (Supplementary Table S1).

When the above significant factors were analyzed for the risk of psychiatric morbidity (with CHQ-12 as the dependent variable) using multiple regression, it was found that the IES intrusion score, DT score, and being female had significant independent effects (Table 5).

## 4. Discussion

The main finding of this study is that hospital workers during the SARS-CoV-2 pandemic had much less psychiatric morbidity, and suffered less psychological impact than hospital workers during the SARS outbreak at the same hospital. Only 21.1% of hospital workers in the current study showed psychiatric morbidity during the SARS-CoV-2 pandemic, which is much lower than the rate observed in hospital workers during the SARS period (75.3%) at the same hospital using the same instrument of assessment (the CHQ) [16,21]. The variations between the two rates can be explained by the high psychiatric morbidity exhibited during the SARS outbreak related to the life-threatening impact of this acute bio-disaster. Other possible explanations for this result are the less severe outbreak of SARS-CoV-2 in Taiwan, the greater experience of hospital workers gained during the SARS outbreak [16], and the proper management strategies of our hospital [32].

Recent studies have reported an estimated prevalence (19.9%) of poor mental health among healthcare workers during the SARS-CoV-2 pandemic [33,34,35,36,37], which is similar to our result (21%). Yang et al. (2021) [38] investigated potential factors related to depression and anxiety in Chinese healthcare workers during the peak of the SARS-CoV-2 pandemic. They found that the prevalence rates of depression and anxiety were 37.8% and 43.0%, respectively. In a meta-analysis, Zhao et al. (2021) compared the prevalence of psychiatric comorbidities in the general population during the SARS and SARS-CoV-2 pandemics. They found that the pooled prevalence of poor mental health during the SARS period (26.6%) was non-significantly different from that during the SARS-CoV-2 pandemic (19.9%) [39]. This finding was contrary to our result. The possible explanations include that, compared to the SARS pandemic in 2003, the SARS-CoV-2 pandemic was relatively well-controlled in Taiwan. In addition, differences in target populations, using diverse research instruments, or including various stages of the disease may also have contributed to the discrepancy. Of note, there is no prior study comparing psychiatric morbidity during the SARS-CoV-2 and the SARS pandemics in the same general hospital. Further follow-up studies are needed to better understand the long-term psychiatric morbidity of SARS-CoV-2.

This might be the first study comparing psychological well-being among workers during the SARS-CoV-2 and SARS pandemic periods in the same general hospital. We found that hospital workers who had SARS experience were more accepting of the risk and responsibility of taking care of SARS-CoV-2 patients than those who had no SARS experience. This result suggests that the hospital workers with SARS experience have more altruism, which is in accordance with the findings of Wu et al. that altruistic acceptance of work-related risks was negatively related to post-traumatic stress symptoms [40]. In addition, we found that hospital workers who had no exposure to SARS were significantly less likely to perceive the danger of becoming infected compared with hospital workers who had exposure to SARS. A possible explanation for this finding is that hospital workers who had no exposure to SARS were younger than those who had exposure to SARS. These young populations might be less fearful because they had no hospital work experience during SARS, or they felt less of a burden of family responsibilities.

The results in the current study indicate that there are no differences in psychological impact, psychological distress, or psychiatric morbidity between hospital workers with or without exposure to SARS. This finding generally supports the “psychological trauma and risk awareness hypothesis”. Prior studies have shown that psychological trauma due to experiencing natural disasters is related to future preparedness and risk awareness [41,42]. This kind of preparedness and risk awareness can help people predict trauma and allow them to more effectively adopt coping mechanisms. Consistent with previous studies, people living in Taiwan during the SARS outbreak in 2003 not only faced severe psychological trauma, but also showed more risk awareness and better preparation for the SARS-CoV-2 outbreak. It is noteworthy that most hospital workers did not primarily care for SARS patients in 2003, and some hospital workers that experienced high psychological stress might have already left the hospitals. Those remaining in this hospital after almost 20 years were those who most likely have better resilience and are now in senior positions. This special group of people may be naturally different from those without SARS experience in terms of age, hospital ranking, and occupational trajectories.

Among 696 hospital workers who had exposure to SARS, we found that the IES intrusion score, the DT scores, and being female were associated factors of psychiatric morbidity. In previous studies, female subjects were shown to be more likely to be depressed or anxious, possibly due to their biological susceptibility or social gender roles [43]. Moreover, this result indicated that the degrees of intrusion or DT were associated with the CHQ; i.e., the IES or DT scores can be predictors of the CHQ. Psychological impact or psychological distress may correlate with psychiatric morbidity.

The present study has several strengths. First, this is the first study to conduct a comparison of the psychological impact and psychiatric morbidity of SARS-CoV-2 and SARS on hospital workers at the same general hospital. Second, the sample size is relatively large. However, the study has certain limitations which need to be considered when interpreting the data. First, despite the relatively large sample size, only about one third of threatened health workers responded. In trauma research, a high response rate is important so as to avoid underestimating the incidence of psychosis, [44]. Second, this was an anonymous survey, making it impossible to compare respondents and non-responders. Participation bias could not be excluded in this study. Third, the use of self-reporting measures instead of diagnostic interviews to assess the incidence of mental illness might lead to false positives. Fourth, there were no queries in our questionnaire about whether the participants primarily cared for SARS patients. The term “SARS experience” in this study reflected whether the participant “worked at the hospital during the SARS outbreak”, but not necessarily whether the participant had “primarily taken care of SARS patients”.

## 5. Conclusions

The psychological impact of SARS-CoV-2 on hospital workers in Taiwan was less than that during the SARS period. In addition, hospital workers who had SARS experience were more accepting of the risk and responsibility of taking care of SARS-CoV-2 pa-tients. Proper management strategies at our hospital and lessons learned from the SARS experience might lead to lower psychiatric morbidity during the SARS-CoV-2 pandemic. Advanced long-term psychological intervention should be suggested if a psychological impact on hospital workers has been identified.

## Figures and Tables

**Table 1:** Comparison of characteristics of respondents during SARS-CoV-2 and SARS periods.

Variable	SARS-CoV-2 *n* = 1816	SARS *n* = 1257
**Gender: *n* (%)**		
Female	1544 (85.0)	1019 (81.1)
Male	272 (15.0)	238 (18.9)
**Age, years: mean (s.d.)**	38.7 ± 10.2	31.8 ± 6.43
**Job title: *n* (%)**		
Doctor	132 (7.3)	139 (11.1)
Nurse	1124 (61.9)	676 (53.8)
Others *	560 (30.8)	442 (35.1)
**Work experience, years: mean (s.d.)**	14.9 ± 10.2	8.5 ± 5.7
**Marital status** **: *n* (%)**		
Married	1002 (55.2)	621 (49.4)
Unmarried	814 (44.8)	636 (50.6)
**Living condition: *n* (%)**		
With family	1385 (76.3)	825 (65.6)
Dormitory/Other	431 (23.7)	432 (34.4))
**Care of SARS-CoV-2 patients/SARS patients: *n* (%)**		
Yes	78 (4.3)	126 (9.9)
No	1686 (92.8)	1022 (81.3)
Not sure	52 (2.9)	122 (9.7)
**Quarantine: *n* (%)**		
Yes	74 (4.1)	79 (6.3)
No	1742 (95.9)	1178 (93.7)

* Others: Including Technician/Administrator.

**Table 2:** Comparison of characteristics of hospital worker respondents who have or have not had SARS experience.

Variable	SARS Experience *n* = 696	No SARS Experience *n* = 1120	t/χ^2^	*p*
**Gender: *n* (%)**			7.88	0.005
Female	571 (82.0)	973 (86.9)		
Male	125 (18.0)	147 (13.1)		
**Age, years: mean (s.d.)**	48.4 ± 6.2	32.6 ± 6.9	−50.80	<0.001
**Job title: *n* (%)**			84.71	<0.001
Doctor	42 (6.0)	90 (8.0)		
Nurse	373 (53.6)	751 (67.1)		
Others *	281 (40.4)	279 (24.9)		
**Work experience, years: mean (s.d.)**	25.3 ± 6.1	8.3 ± 6.0	−58.16	<0.001
**Marital status: *n* (%)**			259.48	<0.001
Married	550 (79.0)	452 (40.4)		
Unmarried	146 (21.0)	668 (59.6)		
**Living condition: *n* (%)**			70.83	<0.001
With family	605 (86.9)	780 (69.6)		
Dormitory/Other	91 (13.1)	340 (30.4))		
**Care of SARS-CoV-2 patients: *n* (%)**			1.24	0.54
Yes	27 (3.9)	51 (4.6)		
No	652 (93.7)	1034 (92.3)		
Not sure	17 (2.4)	35 (3.1)		
**Quarantine: *n* (%)**			48.89	<0.001
Yes	57 (4.1)	17 (1.5)		
No	639 (91.8)	1103 (98.5)		

* Others: Including Technician/Administrator.

**Table 3:** Comparison of psychological impact, distress, and psychiatric morbidity in hospital workers who had or had no exposure to SARS (*n* = 696).

	Yes *n* = 696	No *n* = 1120	B (95% C.I.)	*p*
**IES total scores**	11.6	10.7	0.82(−0.97, 2.60)	0.37
Intrusion	6.0	5.4	0.33(−0.61, 1.27)	0.49
Avoidance	5.6	5.3	0.49(−0.44, 1.41)	0.30
**DT**	2.4	2.1	0.07(−0.24, 0.39)	0.64
**CHQ**				
CHQ scores	1.69	1.44	0.06(−0.39, 0.51)	0.70
CHQ < 3 CHQ ≥ 3	538(77.3) 158(22.7)	895(79.9) 225(20.1)	1.01(0.65, 1.58)	0.97

IES: Impact of Event Scale; DT: Distress Thermometer; CHQ: Chinese Health Questionnaire. Using MANCOVA (Multivariate analysis of covariance) and the controls of gender, age, work experience, living condition, job title, marital status, quarantine, and SARS experience.

**Table 4:** Comparison of the perception of threat in hospital workers who had or had no exposure to SARS.

Item	SARS Experience *n* = 696(%)	No SARS Experience *n* = 1120(%)	B (95% C.I.)	*p*
**I might infect with SARS-CoV-2 because of taking care of SARS-CoV-2 patients.**	351 (50.1)	449 (40.1)	1.13 (0.80, 1.59)	0.49
**I believe the hospital will take good care of me if I infect with SARS-CoV-2 because of my work.**	509 (73.1)	626 (55.9)	0.83 (0.59, 1.19)	0.31
**I have little control over whether I get infected or not.**	203 (29.2)	312 (27.9)	1.45 (1.01, 2.08)	0.043
**I accept the risk of caring for SARS-CoV-2 patients.**	256 (36.8)	286 (25.5)	0.48 (0.34, 0.70)	<0.001
**I think that it is my duty to take care of SARS-CoV-2 patients.**	428 (61.5)	489 (43.7)	0.71 (0.51, 0.99)	0.041
**I think of resigning because of SARS-CoV-2.**	59 (8.5)	153 (13.7)	0.64 (0.38, 1.10)	0.10
**People avoid my family because of my work.**	126 (18.1)	155 (13.8)	1.06 (0.68, 1.65)	0.79
**My workplace had good morale before the SARS-CoV-2 pandemic.**	371 (53.3)	541 (48.3)	0.99 (0.72, 1.38)	0.97
**My workload has increased a lot during SARS-CoV-2.**	385 (55.3)	513 (45.8)	1.11 (0.79, 1.55)	0.55
**I feel more stress at work.**	376 (54.0)	512 (45.7)	0.92 (0.66, 1.28)	0.61
**I think my hospital should provide staff with psychological counseling.**	533 (76.6)	713 (63.7)	0.75 (0.52, 1.08)	0.12

Using logistic regression and the controls of gender, age, work experience, living condition, job title, marital status, quarantine, and SARS experience.

**Table 5:** Risk of psychiatric morbidity in hospital workers who had exposure to SARS: logistic regression analysis.

Item	β	S.E.	Wald	Odd Ratio	C.I.	*p*
DT	0.56	0.06	82.76	1.76	1.56–1.98	<0.001
IES-Intrusion	0.14	0.03	21.25	1.15	1.08–1.22	<0.001
IES-Avoidance	−0.02	0.03	0.38	0.98	0.92–1.04	0.54
Female	0.72	0.33	4.84	2.06	1.08–3.92	0.028

DT: Distress Thermometer; IES: Impact of Event Scale.

## Data Availability

Specific data sets used and/or analysed during the current study are available from the corresponding author on reasonable request.

## Supplementary Materials

The following supporting information can be downloaded at: https://www.mdpi.com/article/10.3390/ijerph19020833/s1, Table S1: Comparison of psychiatric morbidity in clinical characteristics of hospital workers who have SARS experience.
