# Supplementary material for: Psychological Reactions of Hospital Workers to a Pandemic: A Comparison of SARS-CoV-2 in 2020 and SARS in 2003
Source: Int J Environ Res Public Health. 2022 Jan 12;19(2):833. doi: 10.3390/ijerph19020833 (PMC8775670; doi:10.3390/ijerph19020833)
Supplement: Supplementary file 1 [file ijerph-19-00833-s001.zip › ijerph-1489175-Supplementary Table_S1.pdf]

Table S1. Comparison of psychiatric morbidity in clinical characteristics of hospital workers who have SARS experience.

**Variable**	**CHQ<3 N=538 (%)**	**CHQ≧3 N =158(%)**	x^2	**p**
**Gender:**			5.98	0.01
Female	431(80.1)	140(88.6)		
Male	107(19.9)	18(11.4)		
**Age, years:**	48.4±6.2	32.6±6.9	-50.80	<0.001
**Job title:**			8.01	0.09
Doctor	38(7.1)	4(2.5)		
Nurse	285(53.0)	88(55.7)		
Others	215(40.0)	66(41.8)		
**Work experience, years**	25.3±6.1	8.3±6.0	-58.16	<0.001
**Marital status**			0.85	0.36
Married	421(78.3)	129(81.6)		
Unmarried	117(21.7)	29(18.4)		
**Living condition: n (%)**			0.03	0.86
With family	467(86.8)	138(87.3)		
Dormitory/other	71(13.2)	20(12.7)		
**Care of SARS-Cov-2 patients**			4.27	0.12
Yes	19(3.5)	8(5.1)		
Not sure	10(1.9)	7(4.4)		
No	509(94.6)	143(90.5)		
**Quarantine**			0.46	0.50
Yes	42(7.8)	15(9.5)		
No	496(92.2)	143(90.5)		
**IES total scores**	9.41±9.1	18.8±11.3	9.57	<0.001
Intrusion	4.8±4.8	10.1±6.4	9.68	<0.001
Avoidance	4.6±4.9	8.7±5.9	8.78	<0.001
**DT**	1.9±1.6	4.1±1.9	13.52	<0.001

CHQ: Chinese Health Questionnaire; DT: Distress Thermometer; IES: Impact of Event Scale.
